# Control of an Ambulatory Exoskeleton with a Brain–Machine Interface for Spinal Cord Injury Gait Rehabilitation

**DOI:** 10.3389/fnins.2016.00359

**Published:** 2016-08-03

**Authors:** Eduardo López-Larraz, Fernando Trincado-Alonso, Vijaykumar Rajasekaran, Soraya Pérez-Nombela, Antonio J. del-Ama, Joan Aranda, Javier Minguez, Angel Gil-Agudo, Luis Montesano

**Affiliations:** ^1^Departamento de Informática e Ingeniería de Sistemas, University of ZaragozaZaragoza, Spain; ^2^Instituto de Investigación en Ingeniería de Aragón (I3A)Zaragoza, Spain; ^3^Biomechanics and Technical Aids Unit, National Hospital for Spinal Cord InjuryToledo, Spain; ^4^Institute for Bioengineering of Catalunya, Universitat Politécnica de CatalunyaBarcelona, Spain; ^5^Bit & Brain TechnologiesZaragoza, Spain

**Keywords:** spinal cord injury (SCI), brain machine interfaces (BMI), ambulatory exoskeletons, gait rehabilitation, movement intention decoding, electroencephalography (EEG), event related desynchronization (ERD), movement related cortical potentials (MRCP)

## Abstract

The closed-loop control of rehabilitative technologies by neural commands has shown a great potential to improve motor recovery in patients suffering from paralysis. Brain–machine interfaces (BMI) can be used as a natural control method for such technologies. BMI provides a continuous association between the brain activity and peripheral stimulation, with the potential to induce plastic changes in the nervous system. Paraplegic patients, and especially the ones with incomplete injuries, constitute a potential target population to be rehabilitated with brain-controlled robotic systems, as they may improve their gait function after the reinforcement of their spared intact neural pathways. This paper proposes a closed-loop BMI system to control an ambulatory exoskeleton—without any weight or balance support—for gait rehabilitation of incomplete spinal cord injury (SCI) patients. The integrated system was validated with three healthy subjects, and its viability in a clinical scenario was tested with four SCI patients. Using a cue-guided paradigm, the electroencephalographic signals of the subjects were used to decode their gait intention and to trigger the movements of the exoskeleton. We designed a protocol with a special emphasis on safety, as patients with poor balance were required to stand and walk. We continuously monitored their fatigue and exertion level, and conducted usability and user-satisfaction tests after the experiments. The results show that, for the three healthy subjects, 84.44 ± 14.56% of the trials were correctly decoded. Three out of four patients performed at least one successful BMI session, with an average performance of 77.6 1 ± 14.72%. The shared control strategy implemented (i.e., the exoskeleton could only move during specific periods of time) was effective in preventing unexpected movements during periods in which patients were asked to relax. On average, 55.22 ± 16.69% and 40.45 ± 16.98% of the trials (for healthy subjects and patients, respectively) would have suffered from unexpected activations (i.e., false positives) without the proposed control strategy. All the patients showed low exertion and fatigue levels during the performance of the experiments. This paper constitutes a proof-of-concept study to validate the feasibility of a BMI to control an ambulatory exoskeleton by patients with incomplete paraplegia (i.e., patients with good prognosis for gait rehabilitation).

## 1. Introduction

Recovery of lower-limb function in spinal cord injury (SCI) patients is crucial to enhance the independence and quality of life in this population (Ditunno et al., 2008). Two-third of SCI patients are reported as paraplegic (Wyndaele and Wyndaele, 2006), from which a considerable percentage is able to regain certain locomotion function, especially those with low and incomplete lesions (Nene et al., 1996; Scivoletto et al., 2014). Technological advances, such as robotic exoskeletons, have emerged as a valuable option to rehabilitate and restore gait in paraplegic patients beyond traditional means such as crutches, walkers, and orthoses. These robotic systems can range from clinical devices to bioinspired wearable ones. Firstly, weight-suspended robotic systems with a treadmill, such as the Lokomat (Hocoma Medical Engineering Inc, Zurich, Switzerland) and the Gait Trainer (GT II, Rehastim, Berlin, Germany), have demonstrated their rehabilitative efficacy, but they are generally expensive and cannot be used for motor substitution (Wirz et al., 2005). Secondly, robotic walking devices with balance control, such as the REX (REX Bionics Ltd), can be used by people with high SCI (up to C4/5 level), as they completely substitute their gait function, but it may be too bulky and inefficient for those patients who maintain certain balance control. Hence, these systems can be more appropriate for assistive purposes only. Thirdly, ambulatory exoskeletons, such as the ReWalk (Bionics Research Inc) and the H2 (Technaid S.L., Spain), are designed to assist leg movement, but they can only be used by patients with lower-limb weakness who still can maintain balance. These systems are of particular interest, as they can be controlled using assist-as-needed paradigms, which may be more effective than other approaches for rehabilitation and functional compensation of patients with paraplegia and also for stroke sufferers (Pons and Torricelli, 2014).

In this context, there is a growing interest toward the development of robotic devices controlled by brain–machine interfaces (BMI) to assist and rehabilitate gait function (Pfurtscheller et al., 2006; Fitzsimmons et al., 2009; Alam et al., 2014). The contingent link between neural commands and the peripheral feedback given by means of a rehabilitation device can promote neuroplasticity (Mrachacz-Kersting et al., 2012). Whereas, a BMI constitutes a natural interface that provides an easier and more intuitive control of assistive devices (Millán et al., 2010). Non-invasive technologies such, as the electroencephalogram (EEG), constitute a relatively cheap and portable option to build these BMI systems (Wolpaw et al., 2002). The use of brain-triggered rehabilitative technologies is of special relevance for incomplete SCI patients. These individuals can maintain some intact fibers below the injury level, and recent studies have shown how these spared pathways can be reinforced by the continuous association between the activation of the brain during the intention of movement and the stimulation of the paralyzed limbs (Jackson and Zimmermann, 2012).

The closed-loop control of walking exoskeletons using neural commands presents two main challenges. The first one corresponds to the development of robust and reliable BMIs to decode neural signals associated with gait movement intention. In contrast to upper-limb, which is generally the focus of BMI research for motor rehabilitation/restoration of paralyzed patients (Lebedev and Nicolelis, 2006; Millán et al., 2010), decoding of gait has not been so deeply studied. The recording of neural signals during walking might be affected by motion artifacts, which could bias the decoding and lead to misinterpretation of the neural dynamics associated with the movement (Castermans et al., 2014), although there is evidence showing that the influence of these artifacts can be reduced by using carefully designed set-ups (Nathan and Contreras-Vidal, 2016). Nonetheless, recent studies with healthy subjects have shown that EEG neural correlates can be used to decode the gait initiation before it occurs (Jiang et al., 2015; Sburlea et al., 2015) and to distinguish between different walking directions (Velu and de Sa, 2013). However, pathologies like SCI entail a brain reorganization, which may complicate the decoding of motor information (López-Larraz et al., 2015a). Hence, it is important to validate how BMI systems can be applied effectively in these patients. The EEG signals of a paralyzed patient during his/her attempt to move the legs could be decoded without any overt movement and used to trigger the movement of an exoskeleton or prosthesis that assists his/her walking. The current state of the art in non-invasive BMI technology does not allow for precise decoding of fine limb kinematics. Therefore, an accepted approach in the literature is to have a shared control paradigm in which the brain activity is used to trigger the movement of a robot/prosthesis that can autonomously perform a functional task (e.g., walk forward two steps; Millán et al., 2010; Rohm et al., 2013).

The second challenge arises from the complexity of the set-up required to control a device for gait assistance with neural signals. In the recent years, pilot studies have shown how BMIs have been used to control weight-suspended robotic and prosthetic systems (Do et al., 2013; King et al., 2015). Furthermore, robotic exoskeletons with balance control have also been controlled using brain signals (Kilicarslan et al., 2013; Kwak et al., 2015). All these studies are performed with devices that support balance, which minimizes fall risks, and three of them demonstrated successful control with SCI patients (Do et al., 2013; Kilicarslan et al., 2013; King et al., 2015). However, the control of ambulatory exoskeletons with a BMI presents additional issues compared to those systems with balance support. Even if it is used by patients with a relatively good condition (e.g., legs weakness and/or certain degree of balance control), they are required to maintain the balance by holding on to a walker or to parallel bars, and to focus on the intention of motion to command the BMI. This kind of set-up would permit the development of assist-as-needed rehabilitative interventions for such patients, which may lead to higher motor improvements (Cai et al., 2006). Hence, the validation of a BMI to control an ambulatory exoskeleton requires the design of a protocol with special considerations, such as safety, timings and control of patients' fatigue levels during the experiments.

This paper presents an integrated system for the closed-loop control of an ambulatory exoskeleton with a BMI. The exoskeleton works under an assist-as-needed control paradigm, which can be adapted to the capabilities of each patient and assist him/her only to the extent he/she needs. The EEG neural correlates of movement are used to decode the intention of gait initiation, which is used as a volitional control signal for the exoskeleton movement. The feasibility of the proposed system is validated with two sets of experiments. The first experiment shows the viability of the whole set-up with three healthy subjects. The second experiment demonstrates the viability of the system in a realistic clinical environment, involving four incomplete SCI patients. An experimental protocol is proposed to operate the BMI in an ecological set-up, with an emphasis on patients' safety. Decoding performance, exertion levels, and satisfaction and usability scores were measured as indicators of the viability of the system for clinical applications.

## 2. Materials and methods

### 2.1. Participants

Three able-bodied subjects and four SCI patients participated in the study. Demographic data of both the healthy subjects (H) and the patients (P) can be seen in Table 1. The SCI patients were hospitalized at the *Hospital Nacional de Parapléjicos*, in Toledo (Spain), where all the experimentation sessions took place. The inclusion criteria for the patients were: (1) SCI with any lesion level, ASIA C or D with gait prognosis; (2) patients in walking rehabilitation; (3) patient's balance allows standing between parallel bars; (4) no orthostatic complications during standing; (5) upper-limb strength to manage a walker or crutches, and to transfer from the wheelchair to a chair; (6) age between 18 and 60 years; and (7) height 1.50–1.95 m and weight up to 90 kg. The exclusion criteria were: (1) inability to stand in upright position for at least 15 min; (2) any surgery in the previous 3 months; (3) spasticity higher than 3 in the Modified Ashworth Scale (Bohannon and Smith, 1987) in any of the lower-limb muscles; (4) previous/current lower-limb bone fracture; (5) ulcers or sores in areas of contact with the exoskeleton and/or electrodes; (6) previous/current history of cardiovascular disease of any kind or exercise contraindications; (7) upper-limb pain that limits weight bearing on crutches/walker/parallel bars; (8) significant upper/lower extremity discrepancies; (9) uncontrolled autonomic dysreflexia; (10) pregnancy; and (11) cognitive impairment of any kind. The selected patients met all inclusion and no exclusion criteria. All the subjects were duly informed about the study, and all of them gave written consent before the first session. The experimental procedure was approved by the Ethics Committee of the Hospital Complex of Toledo (Spain) (C.E.I.C. 31/02-2014).

**Table 1:** **Demographic information of both groups: healthy subjects and SCI patients**.

**ID**	**Age** **(years)**	**Sex**	**Height** **(meters)**	**Weight** **(kilograms)**	**Injury** **level**	**ASIA**	**Time since** **injury (months)**	**Etiology**
H1	31	Male	1.74	70	–	–	–	–
H2	29	Male	1.77	73	–	–	–	–
H3	29	Male	1.75	74	–	–	–	–
P1	30	Male	1.85	90	L1	C	12	Traumatic
P2	24	Male	1.92	57	L1	C	24	Traumatic
P3	21	Male	1.80	76	T11	C	5	Traumatic
P4	49	Female	1.60	57	T12	C	11	Traumatic

### 2.2. Clinical assessment

We evaluated the clinical condition of the SCI patients before their enrollment in the study. Their injury severity, lower extremity strength, and mobility were measured using a set of clinical tests, according to the standardized ASIA clinical exams (Marino et al., 2003). The lower extremity motor score (LEMS) was used to measure muscle strength, with 5 key muscles examined in each leg: hip flexors, knee extensors, ankle dorsiflexors, long toe extensors, and ankle plantar flexors. The grading system for the muscle strength goes from 0 to 5 (0 = absence of muscle contraction, 5 = normal active movement with full range of motion against full resistance). The cumulative score for the lower extremities ranges between 0 and 50. Modified Ashworth score was used for lower-limb spasticity measurement, ranging from 0 (no spasticity) to 4 (affected part rigid in flexion or extension). Only patient P4 presented a very slight spasticity (score 1) in the right ankle, below the level established in the exclusion criteria. Walking index for spinal cord injury (WISCI II) was used to quantify the degree of assistance required by the patient during normal walking and 10 Meter Walk Test (10 MWT) to assess walking speed (Ditunno and Ditunno, 2001; van Hedel et al., 2005). WISCII II grading system ranges from 0 (patient is unable to stand and/or participate in assisted walking) to 20 (ambulates with no devices, no braces and no physical assistance). According to the recommendations of 10 MWT, walking speed was calculated discarding the 2 initial and the 2 final meters, to only consider walking at a constant speed. Distance (6 m) was divided by the time measured to obtain gait speed (m/s). The values for each patient can be found in Table 2. Walking tests were performed using as little assistance as possible to ensure patient safety.

**Table 2:** **Clinical scores obtained by the patients before the experiments**.

**ID**	**LEMS**	**WISCI II (Technical aid)**	**10 MWT (m/s)**
P1	15	9 (walker and braces)	0.144
P2	20	12 (two crutches and braces)	0.287
P3	17	9 (walker and braces)	0.162
P4	28	15 (one crutch and braces)	0.081

### 2.3. Experimental protocol

The present study was divided into two stages. The first stage aimed at validating the technology under a well-controlled scenario. This was done by performing experiments with healthy subjects and evaluating if the BMI could be effectively used to close the loop and control the ambulatory exoskeleton. The second stage sought to demonstrate that the proposed system and protocol could be safely used in a clinical environment. Experiments with SCI patients were conducted, in which the key point was to measure parameters such as exertion and fatigue levels, as well as usability and satisfaction scales.

**Figure 1:**
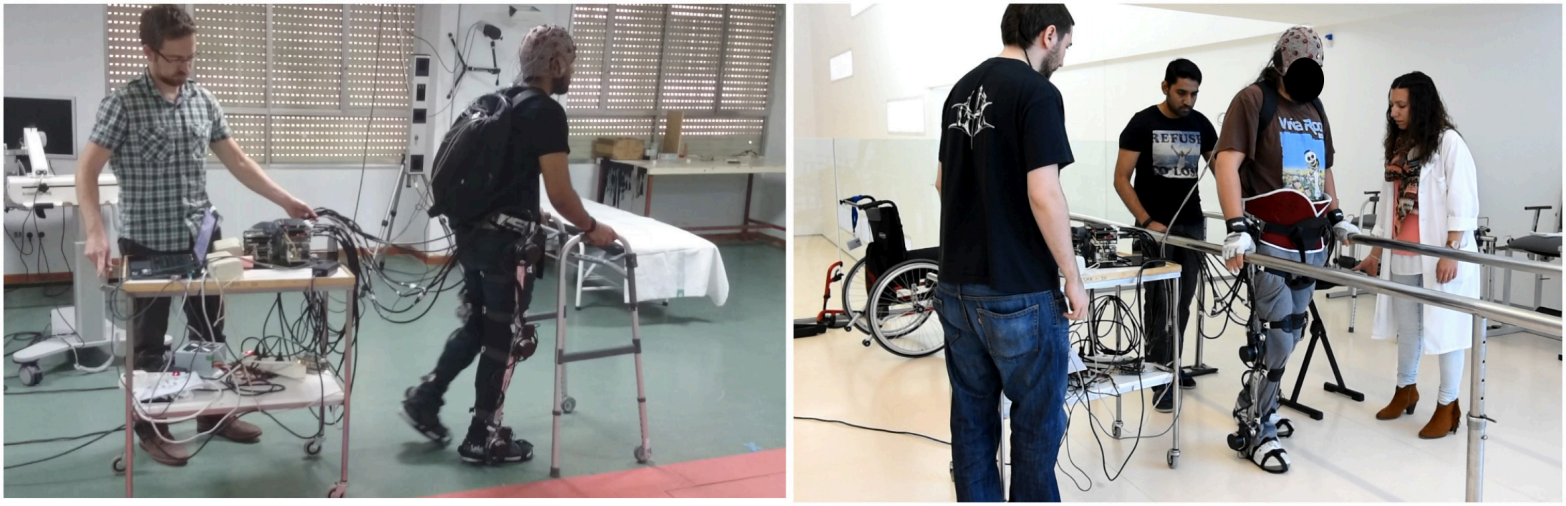
**Snapshots of experimental sessions performed by a healthy subject (left) and a SCI patient (right)**. The EEG cap is connected to the amplifiers that are carried in the backpack. These amplifiers are connected with long cables to a computer placed on the trolley table. The computer processes the EEG signals and sends decoder outputs to the exoskeleton controller, which sends to the joints the commands to move.

The experimental protocol consisted of familiarization sessions and BMI sessions. The experiments with the healthy subjects included the familiarization and the BMI sessions in 1 day. The experiments with the SCI patients required one familiarization session and two BMI sessions in 3 separate days. The set-up included: the EEG equipment (only for the BMI sessions) with the amplifiers in a backpack carried by the subject, the exoskeleton attached to the subject's legs, and a walking aid to help keeping balance (Figure 1). In addition, a trolley table was used to carry the computers that processed the EEG signals and controlled the exoskeleton. Crutches, a walker, and parallel bars were tested as walking aids. Crutches did not provide enough balance control for the patients and were discarded. The walker, which is commonly used in gait rehabilitation by these patients, worked well with the healthy subjects. However, during some preliminary tests with the patients, we realized that they had difficulties to move it while walking with the exoskeleton. Therefore, all the SCI patients performed the BMI sessions using parallel bars, whereas the healthy subjects used the walker. The exoskeleton joints remained blocked whenever it was not in movement in order to partially support patients' weight.

The familiarization sessions allowed the subjects to get used to the protocol timings and the exoskeleton movements. On these sessions, one experimenter triggered the movements of the exoskeleton manually, warning the subject before every movement. For the healthy subjects, these sessions consisted of 5–10 min walking with the exoskeleton. For the patients, the sessions took between 20 and 30 min, in which 2 clinicians monitored every movement and informed the patient about the protocol and how to interact with the exoskeleton. If required, these sessions were repeated until both the patient and the clinicians confirmed that the patient was accustomed to the system, and ready for the first BMI session.

The BMI sessions consisted of screening blocks and closed-loop feedback blocks. Given the nature of the set-up, a cue-guided BMI was proposed, in which the EEG signals were classified asynchronously. Hence, the exoskeleton moved as soon as the intention of movement was decoded, but only during specific periods of time, avoiding sudden and unexpected movements that may result in patients' falls. The participants performed 3 or 4 screening blocks of 20 trials each, which were used to calibrate the BMI decoder. During this screening phase, the participants were standing, wearing the exoskeleton, and holding the corresponding walking aid (i.e., the walker for the healthy subjects, and the parallel bars for the patients). Neither the healthy subjects nor the patients could actually move the legs during the screening blocks (as the exoskeleton joints were blocked). Therefore, in both cases, we consider the action performed as a movement attempt and not as a movement execution. The screening blocks were composed of rest and movement attempt (MA) intervals. The rest intervals had a random duration between 4 and 7 s. An audio cue indicated the start of the MA interval, which lasted 3 s. The participants were instructed to attempt to move their right leg, as if they started walking, immediately after they heard the audio cue. The rest of the time, they were asked to stay relaxed and move as little as possible. During the MA interval, participants were explicitly asked to avoid compensatory movements with the rest of the body, especially with the hip, and to attempt to move their right leg only. The closed-loop feedback blocks were composed of trials with four intervals: (*i*) “Rest,” (*ii*) “Preparation,” (*iii*) “Movement Attempt,” and (*iv*) “Movement.”

The experiments with healthy subjects included 3 blocks of 20 trials each (amounting to 60 trials), in order to acquire enough movements to have a good estimation of the performance of the BMI system. In the experiments with the SCI patients, there was a variable number of trials, and they were asked to reach a distance of 10 m (i.e., the length of the parallel bars), which corresponds to around 20–25 gait cycles. During the “Rest” state (5 s), the subjects were not required to perform any task, but just to relax after the previous trial. After that, a low tone was played, which marked the beginning of the “Preparation” interval (3 s), during which they were instructed to relax and be prepared for the upcoming cue. A high tone denoted the start of the “Movement Attempt” interval (maximum 3 s), in which they were asked to attempt to move their right leg in the same way they had done in the screening blocks. If the BMI detected the intention to move at any time during these 3 s, the system started the “Movement” interval, in which the exoskeleton controller unblocked the joints and moved for one gait cycle: one step with right leg and one with left leg (6 s). Otherwise, after the 3 s, a new trial started in rest state. Supplementary Video 1 shows some trials of subject P1 triggering the exoskeleton with his motor intention.

For safety reasons, every trial required that the experimenter explicitly pressed an *activation* button during the “Rest” or “Preparation” intervals. If that button was pressed, the BMI decoder started sending its outputs to the exoskeleton controller. This would trigger the start of the gait cycle if the patient attempted to move during the “Movement Attempt” interval. If the button was not pressed, the exoskeleton did not move even if the participant attempted to move his/her leg. This mechanism was included in order to avoid starting a movement with the patient being in an unsafe position after the previous gait cycle, and to skip trials to regularly ask the patients about their fatigue levels. When required, the patients could sit for a few minutes to rest, and the trials continued when they confirmed that they were ready.

Due to the complexity of the set-up, the therapist–patient interaction was integral for the correct execution of the BMI experiments. Apart from being the control signal for the exoskeleton, the BMI was used by the therapist to guide the patient during the executions (Pichiorri et al., 2011). A therapist interface was designed to show the experimenter information of the BMI decoder output and the patient's EEG activity in real-time. The experimenter could, for instance, ask the patient to relax if the BMI was detecting movement commands during periods in which the patient should rest (e.g., due to excessive movements required to keep balance) or ask the patient to concentrate further when the BMI was not decoding any movement when they were required. In addition, an option to send manual triggers was included in the therapist interface in order to manually start exoskeleton movements, and so, reduce frustration when the BMI repeatedly failed to decode the movements.

### 2.4. EEG acquisition

The EEG was recorded using a commercial g.Tec system (g.Tec GmbH, Graz, Austria), with 32 channels placed at AFz, FC3, FCz, FC4, C5, C3, C1, Cz, C2, C4, C6, CP3, CP1, CPz, CP2, CP4, FP1, FP2, F7, F3, Fz, F4, F8, T7, T8, P7, P3, Pz, P4, P8, O1, and O2 (according to the international 10/10 system). The ground and reference electrodes were placed on FPz and on the left earlobe, respectively. The EEG was digitized at a sampling frequency of 256 Hz and power-line notch-filtered to remove the 50 Hz line interference. The amplifiers connected to the EEG cap were carried in a backpack by the subject. The amplifiers were connected via long cables to a laptop placed on the trolley table.

### 2.5. Exoskeleton

The exoskeleton used to assist gait was a 6 degrees of freedom wearable lower-limb orthosis with anthropomorphic configuration (Bortole et al., 2015). It included three joints for each leg: hip, knee, and ankle, each of which was powered by a DC motor coupled with a harmonic drive gear. The exoskeleton was equipped with potentiometers and strain gauges to measure the joint angles and the human–robot interaction torques. Its control was conceived to work under an assist-as-needed paradigm in order to make rehabilitation more challenging for the patients. A predefined trajectory, obtained from healthy subjects, was used as the desired gait pattern. The controller updated the stiffness values in real time according to the subject's performance in order to assist him/her just to the extent he/she needed (Rajasekaran et al., 2015). The exoskeleton was connected with long cables to its controller and to the power supply, which were on the trolley table.

### 2.6. EEG-Based movement intention decoder

After recording the screening blocks and before the closed-loop blocks, the BMI classifier was trained to distinguish between rest and movement attempt (MA) classes. The BMI decoder was based on the one proposed in López-Larraz et al. (2014). The decoding of movement attempt was dependent on the combination of two EEG movement correlates: the event-related desynchronization (ERD) of sensorimotor rhythms (Pfurtscheller and Lopes da Silva, 1999) and the movement-related cortical potentials (MRCP) (Shibasaki and Hallett, 2006). Signals from the screening datasets were trimmed down to 7-s trials (−4 to 3 s from the MA audio cue).

#### 2.6.1. Artifact removal

Before training, an automatized procedure based on z-scores was applied to eliminate the trials containing artifacts (López-Larraz et al., 2014). For each trial, the power in delta (1–4 Hz), theta (4–8 Hz), alpha (8–12 Hz), and beta (12–40 Hz) frequency bands, as well as the trial variance and the maximum amplitude were computed. Trials that went over a threshold set at 2.5 standard deviations of the mean in any of these parameters were discarded. Statistical methods like this are especially useful for clinical set-ups as the one presented here, since they do not require human supervision and can be used quickly to eliminate the artifacts before training the BMI (Nolan et al., 2010; Maeder et al., 2012). In principle, this method should be able to remove the most common types of artifacts that can be found in a set-up like this. For instance, analyzing the power in delta band and the signal amplitude may serve to remove low-frequency motion artifacts, whereas analyzing the power beta and signal variance could help to get rid of trials contaminated with EMG artifacts.

For the closed-loop blocks, we considered two options: (1) having an online system to detect EEG artifacts and stopping the BMI every time that it detected one; or (2) considering that the possible artifacts would not highly influence the performance of the system. Given that we trained the BMI with clean trials, the existence of artifacts during test should not deceitfully increase the performance, and at most, they would decrease it. Since we considered that this is not worse than stopping the BMI every time that an online artifact detector detects an artifact, no artifacting was performed during the closed-loop trials.

#### 2.6.2. Feature extraction and BMI training

Features were computed from the clean trials using a 1-s long window with a sliding step of 250 ms. Features corresponding to the rest and MA classes were computed on the [−3, −1] s and [0, 2] s intervals, respectively (with 0 being the time when the high tone indicating the start of the MA interval was played). On these windows, the features were computed as follows:

- ERD features were calculated after applying a small Laplacian filter to the frontocentral, central, and centroparietal EEG channels. After that, a 16th order autoregressive model with a frequency resolution of 1 Hz was used to obtain the power values in the frequency range [7–25] Hz.
- MRCP features were calculated after subsampling the EEG signals to 64 Hz and applying a bandpass filter, [0.1–1] Hz, to them. Then, a common average reference (CAR) was applied to the channels FC3, FCz, FC4, C3, C1, Cz, C2, C4, CP3, CP1, CPz, CP2, and CP4, and their amplitudes were added to the feature vectors.

For each time window, 1192 features were extracted. Sparse discriminant analysis (SDA) was used to select the 30 most discriminant non-redundant features and as a linear classifier (Clemmensen et al., 2011; López-Larraz et al., 2014).

#### 2.6.3. Closed-loop BMI

During the closed-loop blocks, the EEG was processed continuously. A sliding window was computed every 62.5 ms and its features were extracted following the same procedure detailed in Section 2.6.2, and the classifier trained with the screening blocks generated a new BMI output. For each sliding window, the BMI classifier determined if the signal corresponded to rest or to MA classes. In order to ensure a consistent brain activation, the BMI generated a movement trigger when five consecutive windows of MA class were detected (Ramos-Murguialday et al., 2013). If the BMI was active (i.e., if the experimenter pressed the *activation* button during the “Rest” or “Preparation” intervals of a trial, see Section 2.3), the movement trigger was sent to the exoskeleton controller; otherwise, the trigger was not sent. The controller ignored those triggers that arrived during the “Rest” and “Preparation” intervals to avoid starting an unexpected movement, which could make the patients fall. Therefore, on each feedback trial, the exoskeleton moved if the experimenter activated the BMI and the BMI generated a trigger during the “Movement Attempt” interval. In addition, the exoskeleton could also be moved if the experimenter sent a manual trigger during the “Movement Attempt” interval.

### 2.7. Exertion and satisfaction assessments

In order to evaluate the feasibility of the system for clinical applications, the patients were assessed with exertion and satisfaction scales.

The exertion level was assessed three times on each BMI session: before starting (i.e., when the patient was still sitting on the wheelchair), after the screening blocks, and after the closed-loop blocks. The Borg scale was used with values ranging from 6 (“very, very light”) to 20 (“very, very hard”) (Borg, 1970).

After the last BMI session, the patients were asked to evaluate how satisfied they were with the system (i.e., the complete set-up, including the exoskeleton and the EEG system) by using a modified version of the QUEST (Quebec user evaluation of satisfaction with assistive technology) scale (Demers et al., 2002).

## 3. Results

### 3.1. Movement attempt EEG correlates

**Figure 2:**
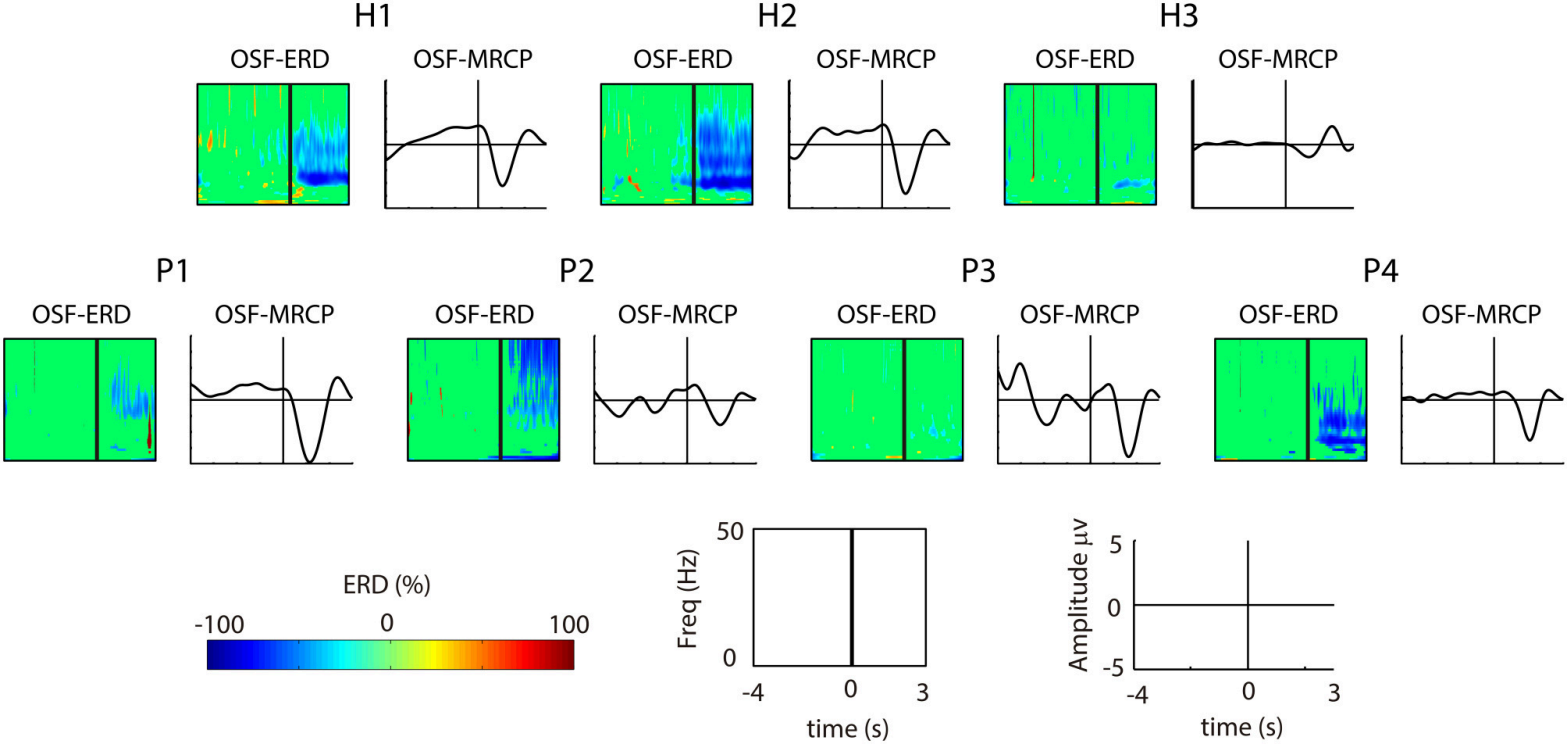
**Significant ERD and MRCP for each subject in the channels obtained by applying optimized spatial filtering**. For each of the 7 subjects (the 3 healthy subjects on top and the 4 patients at the bottom), the left plot shows the ERD, and the right plot shows the MRCP. For the ERD, the *x*-axis correspond to the time interval [−4, 3] s, and the *y*-axis represent the frequency range [1–50] Hz. For the MRCPs, the *x*-axis correspond to the time interval [−4, 3] s, and the *y*-axis represent the MRCP amplitude [−5, 5] μv.

The features used by the BMI to decode the attempts of movement were based on two well-studied EEG correlates: the event-related desynchronization (ERD) of sensorimotor rhythms and the movement-related cortical potentials (MRCP). Figure 2 shows a summary of these correlates computed using the signals recorded in the screening blocks after removing artifactual trials (see Section 2.6.1). For the SCI patients, the screenings from both BMI sessions were combined. Following the methodology proposed in López-Larraz et al. (2014), we used optimal spatial filters (OSF) to visualize the ERD and the MRCP activities by combining the electrodes placed over the motor cortex. Activity recorded on electrodes FC3, FCz, FC4, C3, C1, Cz, C2, C4, CP3, CP1, CPz, CP2, and CP4 was combined with an optimization algorithm, which computed the coefficients that maximized the signal-to-noise ratio of both ERD and MRCPs (Graimann and Pfurtscheller, 2006; Niazi et al., 2011).

Two out of three healthy subjects (H1 and H2) showed strong ERD and MRCP activations, where as H3 showed weak activations of both correlates. For the SCI patients, the ERD patterns of P4 were similar to the ones of H1 and H2; P1 and P2 showed smoother ERD and only in the beta band; and P3 showed no ERD at all. Regarding the MRCPs, P1 and P4 showed similar morphology to H1 and H2, whereas P2 and P3 presented more noisy activity.

### 3.2. BMI performance

**Figure 3:**
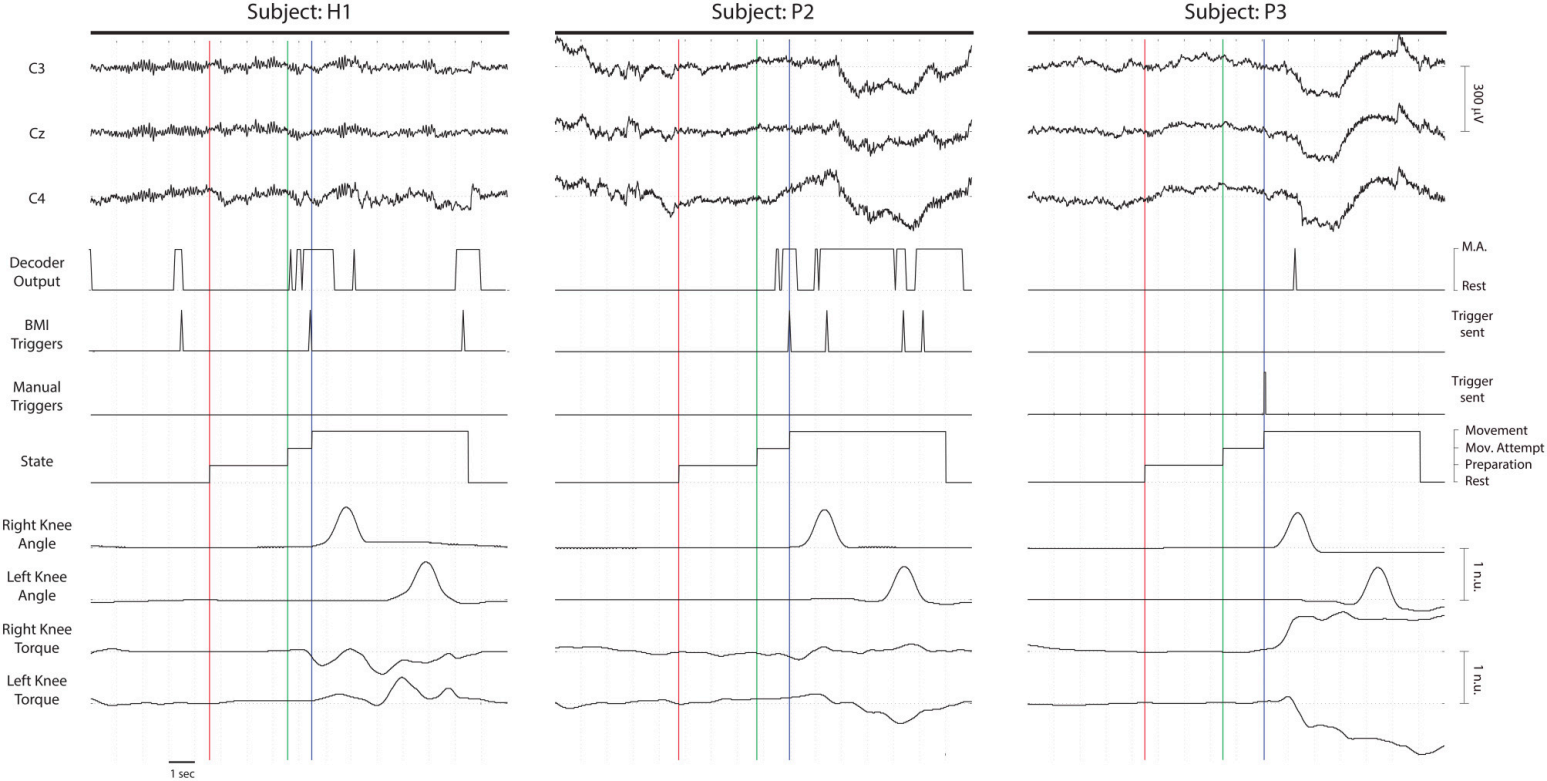
**Time series of three representative trials for healthy subject S1 (left), and patients P2 (center) and P3 (right)**. The three first lines correspond to three EEG channels located over the motor cortex: C3, Cz, and C4. The decoder output indicates the classifier label (Rest or MA) provided for each 1-s window in real time. The BMI triggers were generated only when five consecutive classifier outputs corresponded to MA class, after being in Rest class (they were ignored when generated out of the “Mov.” Attempt period–see left plot). The manual triggers correspond to the movements generated by an explicit command sent by the experimenter (see right plot). The states of the system during a normal trial were: “Rest,” “Preparation,” “Movement Attempt,” and “Movement.” The right and left knee angles (in a normalized scale) indicate the flexion of the knee joints of the exoskeleton. The right and left knee interaction torques (in a normalized scale) measure the forces performed by the subjects in the strain gauges located on the knee joint of the robot. The vertical lines indicate the change of state of the system. The red, green and blue lines correspond to the beginning of the “Preparation,” “Movement Attempt,” and “Movement” phases, respectively.

On average, 14.58% of the trials were rejected before training the BMI decoder (15 ± 1.25% for the healthy subjects and 14.43 ± 2.62% for the patients). Figure 3 shows one representative trial for one healthy subject (H1) and two patients (P2 and P3). For each subject, the following information is shown: data of 3 EEG channels (i.e., C3, Cz, and C4), the decoder output, the triggers generated (i.e., the BMI triggers and the manual triggers), the system states during the trial, the angle of both knees, and the interaction torques measured by the strain gauges of both knees. As can be seen in the left plot, a BMI trigger generated out of the “Movement Attempt” state does not start any movement of the exoskeleton. When it is generated in the appropriate state, the “Movement” period starts, first with the right leg and then followed by the left leg. In the right plot, the BMI did not detect any movement. Instead, the experimenter sent a manual trigger to start the “Movement” phase. The movement of the exoskeleton seems to cause relatively large motion artifacts in the EEG, especially in the patients (center and right plots).

Decoding results for the healthy subjects are presented in Table 3, and for the SCI patients in Table 4. Each of the healthy subjects performed 60 trials (3 blocks of 20 trials each). On average, 84.44% of the trials were correctly decoded, generating a walking movement with the exoskeleton. For the correctly decoded trials of the three subjects, the average time between the auditive cue and the beginning of the exoskeleton movement was 1.07 ± 0.63 s.

**Table 3:** **Decoding results of healthy subjects**.

**ID**	**Number** **of trials**	**Number of** **gait cycles**	**Decoding** **accuracy (%)**	**Decoding time (s)** **mean ± std**
H1	60	53	88.33	1.26 ± 0.53
H2	60	58	96.67	0.90 ± 0.60
H3	60	41	68.33	1.09 ± 0.76

*Given is the number of trials performed, the number of trials in which the BMI decoded the intention of motion (resulting in a walking movement), the decoding accuracy (i.e., the percentage of correctly decoded trials), and the time between the auditive cue and the exoskeleton movement*.

**Table 4:** **Decoding results of SCI patients**.

**ID**	**Session** **number**	**Number** **of trials**	**Number of** **gait cycles**	**Number of** **manual triggers**	**Decoding** accuracy (%)^*^	**Decoding time (s)** **mean ± std**
P1	1	25	21	0	84.00	1.08 ± 0.61
	2	40	20	4	55.56	1.59 ± 0.76
P2	1	2^**^	2	0	100.00	2.69 ± 0.01
	2	28	24	0	85.71	1.54 ± 0.84
P3	1	16	7	2	50.00	1.68 ± 0.67
	2	25	2	14	18.18	0.50 ± 0.52
P4	1	6^***^	5	0	83.33	1.59 ± 0.97
	2	27	23	0	85.19	1.19 ± 0.51

*For each patient and session, given is the number of trials performed, the number of trials in which the BMI decoded the intention of motion (resulting in a walking movement), the number of manual triggers sent by the experimenter, the decoding accuracy (i.e., the percentage of correctly decoded trials), and the time between the auditive cue and the exoskeleton movement*.

* The decoding accuracy was calculated as the number of decoded trials divided by the number of trials in which the experimenter did not send a manual trigger:

*$\%Acc=\frac{\#Gait\;cycles}{\#Trials\;-\;\#Manual\;triggers}\times 100$*.

** *This session was prematurely interrupted due to technical problems with the exoskeleton*.

*** *This session was prematurely interrupted due to temporal restrictions of the participant*.

Given the complexity of the set-up and the unfamiliarity of the patients with the technology, they were asked to attend two BMI sessions. In the first session, which can be considered as a BMI-familiarization session, they were carefully informed about how the BMI system works. Then, they performed the screening blocks and a few closed-loop trials to familiarize with the whole system and protocol. On the second session, patients also performed screening blocks, and subsequently, they started with the closed-loop blocks until reaching a distance of 10 m.

Patient P1 was the only one who performed two successful BMI sessions (i.e., reaching the 10 m distance). In the first one, he achieved a high performance (84%), whereas in the second one, it dropped to 55.56%. For patients P2 and P4, the first session was prematurely interrupted, but both of them performed a successful second session, with more than 85% of decoded trials. For patient P3, performances were low in both sessions, especially in the second one, in which the experimenter had to repetitively use the manual trigger due to the lack of movement attempt commands decoded by the BMI. Notice that the decoding performances presented in Table 4 are computed as the ratio between the number of trials that the BMI correctly decoded and the total number of trials in which the experimenter did not activate the exoskeleton manually. The average decoding accuracy of the successful sessions (i.e., P1 sessions 1 and 2; P2 session 2; and P4 session 2) was 77.61 ± 14.72%, and their average time was 1.35 ± 0.71 s.

Notice that the decoding times reported in Tables 3, 4 correspond to the average time that triggering the movement took with respect to the presentation of the audio cue that indicated the beginning of the “Movement Attempt” interval. Given that different types of delays have an influence on this time (e.g., computational, cognitive, and/or physiological), we also evaluated the average decoding time with respect to the negative peak of the MRCPs. These peaks have been observed to be aligned with the beginning of the muscular activity (Niazi et al., 2011), and hence, may constitute a better indicator of when the subjects started the attempt of movement. For the healthy subjects, the average MRCP negativity appeared 1.02 ± 0.02 s after the auditory cue (Figure 2, first row). Hence, the average decoding time was 50 ms after the MRCP peak. For the 3 SCI patients who performed successful BMI sessions (i.e., P1, P2, and P4), the MRCP negativity appeared on average 1.37 ± 0.20 s after the cue. Therefore, the average decoding time for the patients was 20 ms before the occurrence of their MRCP peak.

Although the proposed protocol impeded that the exoskeleton could start moving during “Rest” and “Preparation” time intervals, we analyzed the offline movement triggers that were generated by the BMI in those intervals. To that end, we calculated the number of trials in which, at least, one movement trigger was generated during non-movement periods. For the healthy subjects, movement triggers were generated in 52.22 ± 16.69% of the trials during the “Preparation” interval, and in 66.67 ± 13.02% of the trials during the “Rest” interval. For the successful sessions with the patients, movement triggers were generated in 40.45 ± 16.98% of the trials during the “Preparation” interval, and in 63.42 ± 14.15% of the trials during the “Rest” interval. We performed 3 statistical tests to compare the percentages of trials with correct triggers during the “Movement Attempt” period (true positives) and with erroneous triggers generated during “Rest” (false positives during “Rest”) and “Preparation” (false positives during “Preparation”) periods. For each measure, a single vector was generated by concatenating the percentages of the three healthy subjects and the four successful sessions of the patients, and paired Wilcoxon signed rank tests were used to measure significant differences. The number of true positives was significantly higher than the number of false positives generated during the “Preparation” interval (*p* < 0.05), although true positive was not significantly higher than the number of false positives during the “Rest” interval (*p* = 0.08). In addition, the number of false positives during the “Rest” interval was significantly higher than the ones generated during the “Preparation” interval (*p* < 0.05).

### 3.3. Features

**Figure 4:**
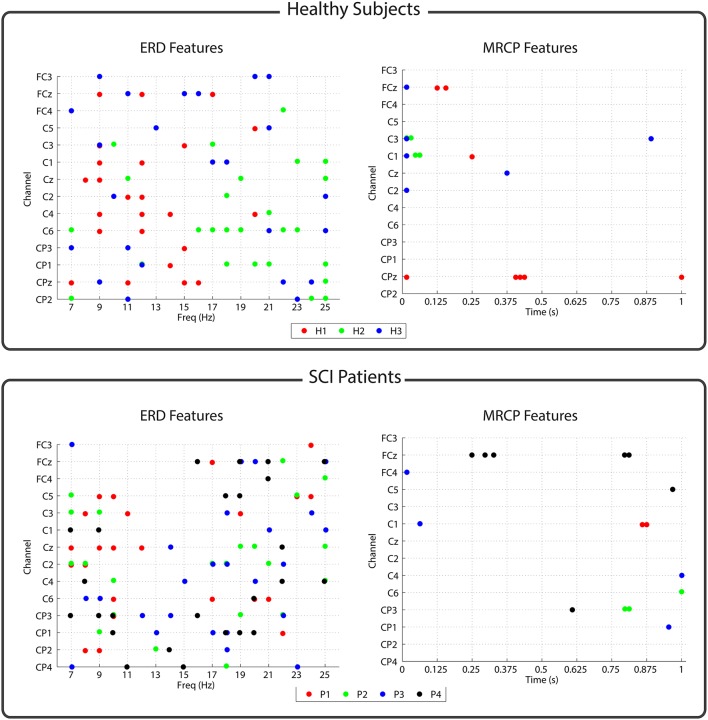
**Features selected by the SDA algorithm for each subject**. Upper/lower panel corresponds to the features of the healthy subjects/patients. The left part of each panel shows the ERD features that were selected as channel-frequency pairs. The right part of each panel shows the MRCP features that were selected as channel-time pairs.

As an automatic feature selection algorithm was used during the experiments, we performed a *post-hoc* analysis to evaluate the selected features for each participant. Figure 4 shows the features selected by the SDA algorithm for the healthy subjects and the patients. For the patients, the reported results correspond to the second BMI session. As can be seen, more frequency (ERD) than temporal (MRCP) features were consistently selected in both groups. For the ERD features, pairs in the whole channel-frequency space were selected for all the subjects. Central and centroparietal electrodes were more consistently selected than frontocentral ones. In addition, certain subjects showed a higher density of features allocated in specific regions of the channel-frequency map. For instance, for subject H1, more features were selected in the alpha band (8–12 Hz), whereas for subject H2, there were more in beta band (15–25 Hz). MRCP features were more scarcely chosen by the algorithm, although in all of the participants, some of them were selected.

### 3.4. Exertion and satisfaction assessments

Table 5 shows the values of the Borg scale given by each patient on each session. At the beginning of the session, all the patients reported the minimum exertion level. As described above, each patient performed 3 or 4 screening blocks (each of which lasted around 3 min), resting between blocks as long as they required. After these screening blocks, all the patients reported an increase between 3 and 5 points on their exertion level. Subsequently, they started the closed-loop blocks, in which they walked a maximum of 10 m with the exoskeleton. Here, exertion levels slightly increased for 1 or 2 points in most cases. The only exceptions were P2 in session 1 (which just performed 2 gait cycles due to technical problems), who did not report any increase after screening; and P3 in session 2, who increased 5 points. None of the values of the Borg scale exceeded 17, which is considered as the limit value for maximal exertion.

**Table 5:** **Results of the Borg scale**.

**ID**	**Session**	**Pre session**	**After screening**	**Post session**
P1	1	6	11	12
	2	6	10	11
P2	1	6	11	11
	2	6	9	10
P3	1	6	11	13
	2	6	10	15
P4	1	6	10	11
	2	6	10	11

*For each patient and session, the exertion levels were measured when the patient arrived (Pre session), after performing the screening blocks (After screening), and at the end of the session (Post session). The values of this scale range from 6 (“very, very light”) to 20 (“very, very hard”)*.

The results of the satisfaction test are presented in Table 6. The highest score for the QUEST scale was obtained in the questions about safety and security, and easiness of use (4.25 on average), whereas the lowest was obtained by the question about comfortability (2.5 on average).

**Table 6:** **Results of each patient on the modified QUEST scale**.

**Question**	**P1**	**P2**	**P3**	**P4**	**Mean**
How satisfied are you with:					
1. the **dimensions** (size, height, length width) of the device?	4	2	4	1	2.75
2. the **weight** of the device?	3	3	5	1	3
3. the **ease in adjusting** (fixing, fastening) the parts of the device?	2	4	4	2	3
4. how **safe and secure** the device is?	5	4	5	3	4.25
5. the **durability** (endurance, resistance to wear) of the device?	3	3	4	4	3.5
6. how **easy** is it to use the device?	5	3	5	4	4.25
7. how **comfortable** the device is?	3	2	4	1	2.5
8. how **effective** the device is to solve the problem for which you are using it?	4	4	4	3	3.75
9. What is your level of satisfaction with the device in general?	5	2	4	3	3.5
**Total:**	34/45	27/45	39/45	22/45	30.5/45

*1, Not satisfied at all; 2, Not very satisfied; 3, More or less satisfied; 4, Quite satisfied; 5, Very satisfied*.

### 3.5. Exoskeleton adaptive control

An important feature of the system introduced in this paper was that, since we used an ambulatory exoskeleton, we could introduce an assist-as-needed control paradigm, which may serve to make the rehabilitation interventions more challenging (Pons and Torricelli, 2014). Although it was not one of the main goals of this study, we measured the degree of assistance that the control strategy provided to healthy subjects and patients. The gait assistance for the healthy and SCI individuals is provided based on the adaptive control model presented in Rajasekaran et al. (2015). The adaptive control applies an efficient stiffness to each joint, which is computed based on the interaction torques and position error of each joint. Hence, the assistance level is defined based on the variation in the stiffness parameter. For healthy subjects, the stiffness values for hip, knee, and ankle were 60 ± 4 Nm/deg, 60 ± 2 Nm/deg, and 60 ± 5 Nm/deg, respectively. For the SCI patients, the stiffness for hip, knee, and ankle were 80 ± 2 Nm/deg, 82 ± 5 Nm/deg, and 80 ± 5 Nm/deg, respectively.

## 4. Discussion

The present study proposed a novel system BMI with an ambulatory walking exoskeleton. Its feasibility has been shown with experiments performed by three healthy subjects and four spinal cord injury (SCI) patients. The BMI decoded the brain activity related to the intention of movement and sent the commands to the robotic system. The robot moved for two steps (one with the right and one with the left leg), using an assist-as-needed strategy, which assisted the patients only to the extent they needed. The degree of assistance was shown to be higher for the SCI patients, which could not perform the movement autonomously, compared to the healthy subjects, who did not have to make a high effort to follow the exoskeleton during its walking movements. This is, to the best of our knowledge, the first study in which a walking exoskeleton with no weight or balance support is controlled by incomplete SCI patients with a BMI. This may serve as a proof-of-concept for future studies in which a larger sample could allow the assessment of the rehabilitative effects of this type of BMI-controlled ambulatory exoskeletons.

In spite of the fact that EEG-based BMI technology is still far from being a practical control system for gait-assistive devices, its applicability for rehabilitation within clinical environments may be available in the next few years. In fact, its use has a great potential for rehabilitation of incomplete SCI patients. In these patients, there are still some pathways communicating the brain and the limbs, which can support some degree of functional recovery (Jackson and Zimmermann, 2012). The persistent causal relationship between the brain activation during the intention of movement and the stimulation of the limb (e.g., with an exoskeleton or electrical stimulation) has demonstrated its viability to induce Hebbian plasticity in animal studies (Jackson et al., 2006). Most BMI studies aiming to control rehabilitative devices are focused on the upper-limb, and they have demonstrated the possibility of controlling robotic systems (Gomez-Rodriguez et al., 2011; Ramos-Murguialday et al., 2012, 2013; Bhagat et al., 2016) or functional-electrical stimulation (FES) (Pfurtscheller et al., 2003; Rohm et al., 2013). For the lower-limb, there are less examples in the literature of closed-loop non-invasive brain-controlled systems. The group led by Dr. Nenadic (University of California, USA) demonstrated the BMI-based control of weight-suspended robotic (Do et al., 2013) and FES (King et al., 2015) gait-assistance systems. Furthermore, two recent studies have used brain signals to control the REX (REX Bionics Ltd), a robotic system that provides weight and balance support for patients with a high degree of gait disability (Kilicarslan et al., 2013; Kwak et al., 2015). The system proposed in this paper utilized an ambulatory exoskeleton, which does not rely on any weight support beyond the walker or the parallel bars. Hence, our approach targets patients with incomplete and lower lesions, who can walk short distances with the help of crutches or walkers, and who are the ones with best prognosis for gait rehabilitation (Nene et al., 1996; Scivoletto et al., 2014).

During the screening blocks, the exoskeleton joints were blocked, and hence, even the healthy subjects could just perform the attempt of movement, and not an overt movement. The EEG correlates of these movement attempts, namely the ERD and the MRCPs, were used as features to train the BMI decoder for the closed-loop blocks. The combination of both activation patterns has been shown to be beneficial to improve the movement intention decoding (Ibáñez et al., 2014; López-Larraz et al., 2014), as it may prime the use of features from the signals with higher degree of activation (e.g., P2 which showed a significant ERD, but an MRCP with a low amplitude, see Figure 2). In a *post-hoc* analysis, we observed that, in general, ERD features were more consistently selected by the automatic feature selection algorithm than MRCP features. There is certain controversy with regard to how these correlates can be affected by motion artifacts during walking (Castermans et al., 2014; Nathan and Contreras-Vidal, 2016). In fact, we observed that slow oscillations were present in some trials during the movements of the exoskeleton, especially in the patients (see Figure 3, center and right plots). Anticipating this, we preferred to train the decoder using signals recorded during the attempt of movement and not during actual movement. To minimize artifacts in the training data, we used an automatic artifact rejection method that eliminated contaminated trials (e.g., by slow movement oscillations or EMG artifacts). Moreover, we carefully instructed all the participants to only perform the attempt of movement of their right leg, avoiding compensatory movements with the rest of the body. However, the attempt of movement of a paralyzed limb is a complex task, especially for the paraplegic patients, who had to concentrate on keeping balance at the same time. Therefore, in the protocol we proposed, it is not possible to guarantee that they were only attempting to move the right leg, as they may also be activating arm or trunk muscles, which normally help them to move their legs during their normal walking rehabilitation. This means that the brain activations we measured in the patients may include not only the attempt of movement of the leg but also compensations of other body parts. Comparison of the brain activation patterns between the groups of healthy subjects and patients was out of the scope of this work. Firstly, because the number of participants was small to perform an accurate neuroimaging study, and secondly, because the set-up and task performed by each group were slightly different (i.e., walker and attempt of movement of a healthy limb for the healthy subjects; parallel bars and attempt of movement of a paretic limb for the SCI patients). Nonetheless, in terms of the brain activations, we observed that the subjects who presented weaker brain activations during the attempt of movement were the ones with poorer decoding performances: H3 and P3. Three of four patients had long-term injuries, between 11 and 24 months. Curiously, these three patients were the ones with best decoding performances, showing similar values to healthy subjects, in contrast to P3, who had a 5-months injury but could not control the BMI correctly.

SCI modifies the brain activity related to movement (Müller-Putz et al., 2007; Castro et al., 2013; López-Larraz et al., 2015a). Although there is a large body of literature showing that movement intention can be decoded from EEG signals on these patients (Pfurtscheller et al., 2003; López-Larraz et al., 2012; Rohm et al., 2013; King et al., 2015), among others, this neuroplastic process may affect the reliability of rehabilitative and assistive BMI systems to be used by SCI patients. The heterogeneity of incomplete lesions will probably result in significant differences in the neural reorganization processes followed by the patients' brains (Freund et al., 2013). Hence, an interesting research pursuit for the next years will be the characterization of the brain changes following SCI, which will set the basis to personalize the systems to improve their applicability.

Regarding the decoding algorithms to detect the movement intention, extensive research is being conducted toward the optimization of signal processing and classification techniques to increase BMI performance (Bashashati et al., 2015; López-Larraz et al., 2015b). The procedure used in this work has been previously used to decode movements of the upper-limb with incomplete tetraplegic patients (López-Larraz et al., 2014), and the accuracies that we achieved were similar to other recent studies detecting gait initiation (Jiang et al., 2015; Sburlea et al., 2015). Five consecutive windows with the classifier indicating a motor attempt output were required to start the movement. Although it implied a constant delay of 250 ms with respect to the first output, this mechanism was used to ensure a consistent brain activation and not just a spurious change that may lead to false positive activations There is evidence stating that short delays are beneficial to facilitate plasticity in the brain (Mrachacz-Kersting et al., 2012), and recent studies have worked on developing methodologies to anticipate movements or to decode them with a short latency (López-Larraz et al., 2014; Xu et al., 2014; Jiang et al., 2015; Sburlea et al., 2015). However, these studies require a precise measurement of the time instant when the movement starts in order to calibrate the BMI. Due to the typology of the patients recruited for this study, we could not have a reliable signal to identify the movement onset, even measuring the muscle activity with electromyography. Therefore, we considered that the delay induced by our methodology to detect the movement intention could be acceptable to control the exoskeleton, as a similar procedure has demonstrated its efficacy for neurorehabilitation of stroke patients (Ramos-Murguialday et al., 2013). In a *post-hoc* analysis, we compared the latency of the decoder with respect to the MRCP peak negativity, and observed that the difference was minimal (+50 ms for the healthy subjects and −20 ms for the patients). MRCP negativity is, in general, aligned to the onset of muscular activity during voluntary movements (Niazi et al., 2011). Although it should not be used as a robust measurement of decoding latency, this metric allowed us to estimate how this time would be with respect to the EMG activations. In any case, we consider that more investigation is required to evaluate how different trade-offs (e.g., priming temporal precision, or guaranteeing consistent brain activation) can affect BMI performance and rehabilitative outcomes.

A cue-guided BMI protocol was proposed so that the participants always knew in which phase of the trial they were. Furthermore, the shared control strategy implemented implied that the exoskeleton movements were only enabled in specific time periods (i.e., the “Movement Attempt” intervals), ensuring that no unexpected movements could happen when the patients were not ready. The BMI analyzed the brain signals continuously, meaning that the movement triggers were generated asynchronously at any time during the trial. If these triggers were generated during the “Movement Attempt” interval, then the gait cycle gets started, and otherwise, they were ignored. The offline analysis revealed that movement triggers were generated in a high percentage of trials during the “Rest” (i.e., accommodation period, in which subjects could move and rest) and “Preparation” (i.e., relaxation period preceding the “Movement Attempt”) intervals. The movement attempts were correctly decoded in 84.44 ± 14.56% and 77.61 ± 14.72% of the trials, for healthy subjects and patients, respectively. These percentages were significantly higher than the number of trials with movement triggers generated during “Preparation” interval (52.22 ± 16.69% and 40.45 ± 16.98%), but not greater than the trials with triggers generated during “Rest” interval (66.67 ± 13.02% and 63.42 ± 14.15%). Notice that in this latter period, the participants sometimes moved to accommodate their position after the previous trial. These numbers suggest that the use of shared-control strategies designed to avoid non-desired robot movements, like the one proposed in this paper, can facilitate the integration of BMI technology in clinical set-ups (Rohm et al., 2013). These take special relevance in gait rehabilitation therapies, in which keeping the balance may cause movements during rest periods that lead to more false positives.

The proposed approach aims at decoding the intention of motion to generate a functional movement (i.e., a gait cycle) with the exoskeleton. The repetitive association between the brain activation related to the motion intention and the peripheral feedback may reinforce the corticospinal circuits and promote Hebbian synaptic plasticity (Jackson and Zimmermann, 2012). An alternative and interesting approach for future would be to develop a system that continuously controls the exoskeleton movements instead of decoding the intention of movement and triggering a predefined trajectory. This should be the preferred strategy for assistive devices. Presumably, it may also improve rehabilitative effects by a more consistent association between paired firing of neurons, which may accelerate the neuroplastic changes (Jackson and Zimmermann, 2012). To date, promising results toward the continuous control of gait rehabilitations devices with EEG have been shown in preliminary studies with weight-suspended and self-supported systems (Do et al., 2013; Kilicarslan et al., 2013; King et al., 2015; Kwak et al., 2015). However, there are still several issues that need to be improved before the effective implantation of this technology in clinical practice, such as signal processing techniques or artifacts management (Castermans et al., 2014; Nathan and Contreras-Vidal, 2016). Some of these issues are of especial relevance when using ambulatory exoskeletons, which require extra considerations to improve safety, as the control strategy implemented to deal with possible false positives. Furthermore, the rehabilitative effects of each type of intervention still have to be quantified with clinical studies. For now, the development and improvement of novel interventions, like the one proposed here, aim at increasing the number of possible interventions to rehabilitate gait. Eventually, the clinicians will be in charge of evaluating the risks and benefits to recommend the most suitable interventions for each specific patient (e.g., BMI continuous control of prosthesis, or BMI-triggered predefined movements, as the one proposed in this study).

The main goals of this study were to test the BMI-exoskeleton system and to propose a methodology that may be followed in future studies combining BMI and ambulatory exoskeletons. Therefore, the design of the protocol was a key point to be able to validate the technology and the set-up with the patients. We observed that a familiarization session with the exoskeleton was necessary before the BMI session to allow the participants to get used to the dynamics of the system. While for the healthy subjects, a 5–10 min familiarization was enough, the patients required a specific session due to their poor balance and walking capabilities. Several issues have to be tailored for each patient depending on his/her capabilities, like the cadence or the distance between joints. Rehabilitative devices such as the one used in this study have to prioritize patient's safety. The recruited patients were capable of standing and ambulating without the aid of a harness, which required a complex set-up and additional safety measures to avoid falls. In this respect, the experimenter played an important role by controlling the system, which functioned as a “dead-man's” switch. This means that at the beginning of every trial, the experimenter had to make sure that the patient's legs and feet position were appropriate and ask him/her if he/she was ready for another step. This methodology tried to imitate the procedure followed by physiotherapists for patients in the early stages of gait rehabilitation with rigid leg orthoses. In addition, the experimenter had to monitor the EEG signals and the BMI output in order to verify that everything was correct and guide the patients when they lost concentration or generated artifacts by excessive movements. The use of BMI technology to provide the therapist with objective information about the patient's performance has been stated to be very important for the implantation of this type of systems in clinical environments (Mattia et al., 2013; Asín Prieto et al., 2014). We consider that the good results achieved in this study were, in part, due to the therapist–patient interaction that was augmented—thanks to the designed therapist interface.

The fatigue level of the patients was also continuously monitored, and they could ask for a pause whenever they wanted to relax for some minutes. This is, probably, the reason of the relatively low levels of exertion shown by the patients when asked at the end of the session. In terms of usability and satisfaction, the patients were not so positive as we had expected, but they provided very useful information that encourages the authors to continue working to improve the wearability and comfort of the system. Based on the high scores of the questions about safety and security, we believe that the security measures implemented in this study succeeded and they could be applied for future studies. In general, two of the patients (P1 and P3) were rather satisfied with the system, whereas the other two (P2 and P4) reported that several issues could be improved, especially those related with comfort and wearability of both the exoskeleton and the EEG system. Nonetheless, all of them appraised the potential of the combination of both technologies. Patients found the use of the conductive gel for the EEG recording as one of the main inconveniences, which is in line with other works (Rupp, 2014). We believe that the potentiality of dry electrodes will have to be explored for future prototypes to be used with patients in clinical practice. Another important limitation of the system is the difficulty to wear the exoskeleton, as at least two people had to assist the process. This is an important factor to improve for future designs of exoskeletons with clinical applicability. Ideally, the patients should be able to wear the exoskeleton by themselves. All of them reported their willingness to keep participating in rehabilitation interventions with this technology, which is a promising sign for the future of BMI-based gait rehabilitation.

It is important to mention that due to the nature of the study and the typology of patients recruited, the number of trials recorded with these patients was small. Nonetheless, the BMI performances are in line with similar works performed for the upper- and lower-limbs. The main contribution of this work is to validate the feasibility of a BMI system and protocol to control an ambulatory exoskeleton for gait rehabilitation without weight or balance support. However, this preliminary study does not allow yet to draw conclusions about the rehabilitative potential of this technology. Further experiments with a larger population of patients and with a larger number of sessions will be required to evaluate if the therapeutic potential of BMI for gait rehabilitation approaches the traditional therapies. New challenges appear when it comes to pursuing clinical trials integrating this kind of novel technologies with patients. Adapting the systems for different pathologies and personalization of the technology will be of paramount importance for the use of BMI systems in clinical practice (Rupp, 2014). In addition, standardizing metrics to evaluate system performances and clinical outcomes will facilitate the validation of BMIs for their implantation in rehabilitative centers (Venkatakrishnan et al., 2014).

## Author contributions

Conceived and designed the experiments: EL, FT, VR, AD, JA, LM. Performed the experiments: EL, FT, VR, SP. Analyzed the data: EL, FT. Drafted the manuscript: EL, FT, VR. Revised the manuscript: EL, FT, VR, SP, AD, JA, JM, AG, LM.

### Conflict of interest statement

The authors declare that the research was conducted in the absence of any commercial or financial relationships that could be construed as a potential conflict of interest.
